# The Influence of Two-Dimensional Organization on Peptide Conformation**

**DOI:** 10.1002/anie.201408971

**Published:** 2014-11-20

**Authors:** Simon J White, Steven D Johnson, Mark A Sellick, Agnieszka Bronowska, Peter G Stockley, Christoph Wälti

**Affiliations:** Astbury Centre for Structural Molecular BiologyUniversity of Leeds, Leeds LS2 9JT (UK); Department of Electronics, University of YorkYork, Y010 5DD (UK); School of Electronic and Electrical Engineering, University of LeedsLeeds LS2 9JT (UK); Heidelberg Institute for Theoretical Studies gGmbH69118 Heidelberg (Germany)

**Keywords:** coiled coil, molecular crowding, circular dichroism, peptide conformation, surface analysis

## Abstract

Molecular crowding plays a significant role in regulating molecular conformation in cellular environments. It is also likely to be important wherever high molecular densities are required, for example in surface-phase studies, in which molecular densities generally far exceed those observed in solution. Using on-surface circular dichroism (CD) spectroscopy, we have investigated the structure of a synthetic peptide assembled into a highly packed monolayer. The immobilized peptide undergoes a structural transition between α-helical and random coil conformation upon changes in pH and ionic concentration, but critically the threshold for conformational change is altered dramatically by molecular crowding within the peptide monolayer. This study highlights the often overlooked role molecular crowding plays in regulating molecular structure and function in surface-phase studies of biological molecules.

Molecular crowding plays an essential role in the assembly and stability of many macromolecular complexes, for example, α-synuclein fibrils[1] and the HIV-1 capsid protein.[2] Similarly, the activity of enzymes, such as superoxide dismutase for catalase,[3] is strongly affected by molecular crowding. A comprehensive understanding of molecular structure and function must therefore consider the effects of local concentrations, particularly in environments that exhibit very high molecular densities.

High-density molecular environments are observed and exploited frequently in systems involving the integration of biomolecules with inorganic surfaces. Many biophysical analytical techniques, such as surface plasmon resonance (SPR), the quartz crystal microbalance with dissipation monitoring (QCM-D), and a wide range of emerging technologies, notably in vitro diagnostics, tissue engineering, and biomolecular electronics, all require the immobilization of biological molecules onto a surface, often at very high surface densities. For example, alkanethiolates on gold exhibit typical surface densities of up to 4×10^14^ molecules per cm^2^,[4] equivalent to a molecular concentration within the monolayer in excess of 2 m for mercaptohexadecanoic acid and significantly higher than typical cellular concentrations. To date, research into high-density biomolecular layers has focused on the role of molecular crowding in regulating interactions between the immobilized molecules and solution ligands. For example, within high-density nucleic acid monolayers the hybridization efficiency and specificity is influenced by electrostatic and steric crowding effects.[5] Similarly, the surface density of immobilized proteins can regulate the kinetics and thermodynamics of protein–ligand interactions.[6] Despite the importance of surface-immobilized protein and peptide molecular systems, little is known about the local arrangement of molecules on the surface or how crowding within the molecular layer influences molecular conformation.

Here, we provide critical insight into the influence of molecular crowding on the two-dimensional organization and molecular conformation of a model peptide in a self-assembled monolayer. Using on-surface CD, we show that an α-helical secondary structure can be induced and stabilized by intermolecular interactions within the densely packed peptide layers. Our findings not only provide insight into molecular crowding effects that occur in high-molecular-density environments, such as within the cell, but also highlight the potential role that molecular crowding can play in many current and emerging technologies involving surface immobilized biomolecular systems.

Measurements of molecular conformation in solvated native protein films are challenging and conformational integrity is typically inferred from measurements of protein activity. However, a range of noncrystallographic biophysical tools, such as IR spectroscopy[7] and X-ray and neutron scattering techniques,[8] are now available for characterizing the conformation and conformational changes of solvated native proteins, with CD spectroscopy being the most commonly applied method. Although conventionally applied to proteins in solution, the technique has recently been developed to study surface-immobilized proteins.[9] Despite significant reduction in signal intensity for surface-phase measurements, the relative accuracy and sensitivity are sufficient to resolve the secondary structure, for example the technique has been used to monitor unfolding of a protein with 60 % α-helical content.[9b]

Here, we use CD to study the conformation of surface-tethered peptide monolayers. We have investigated a 30-residue synthetic peptide, BASE-C,[10] which includes a C-terminal cysteine (the only cysteine in the peptide) for oriented and covalent attachment to thiol-reactive surfaces. We have previously shown that surface-immobilized BASE-C still forms heterodimeric coiled-coil complexes with the corresponding ACID peptide.[11] The charged lysine residues at positions e and g of the heptad destabilize the assembly of coiled-coil homodimers at physiological pH. Solution-phase CD spectra (Figure 1) clearly demonstrate this effect. Above pH 9, the deprotonation of the lysine residues reduces electrostatic repulsion between BASE-C peptides, resulting in the assembly of stable coiled-coil homodimers. At pH 11, the ratio of the CD minima at 222 nm and 208 nm, *Θ*_222_/*Θ*_208_, approaches 1, suggesting that the peptides assemble predominantly into stable coiled-coil homodimers.[12] Electrostatic effects dominate below pH 9 and BASE-C exists as an isolated, random-coil peptide. These electrostatic interactions can be screened by increasing ionic strength (Figure S1 in the Supporting Information, SI). From the *Θ*_222_/*Θ*_208_ versus NaCl concentration data (Figure 1 inset), it can be seen that BASE-C peptides form pronounced helical conformations at pH 3 at NaCl concentrations ≥0.5 m, and that the stable coiled-coil homodimer assembly dominates at 2.5 m NaCl. We note that the ionic strength of the buffers ranges from 30 mm (pH 9) to 34 mm (pH 11), which is significantly lower than the NaCl concentrations required to screen electrostatic repulsion.

**Figure 1:**
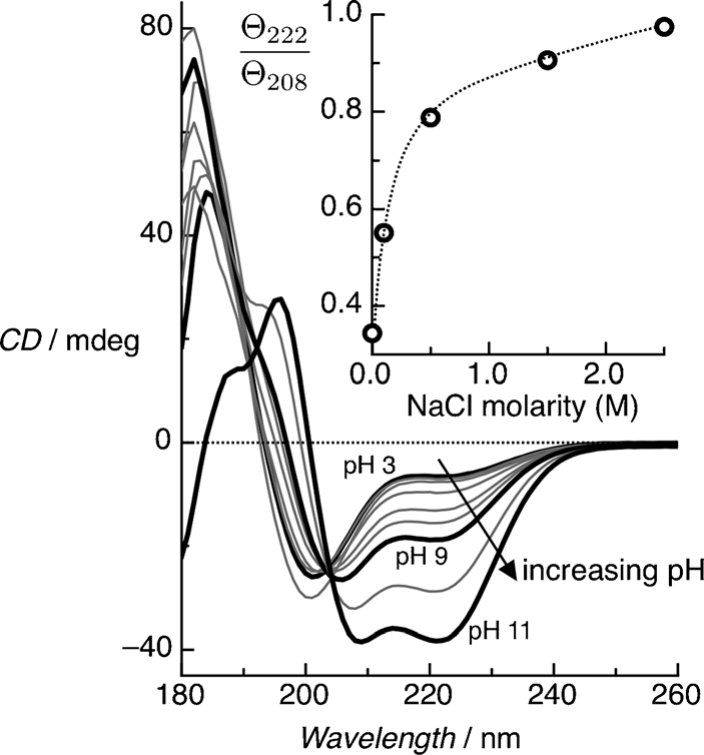
Solution-phase CD spectra for the BASE-C peptides as a function of pH. Pronounced CD minima at 222 nm and 208 nm, characteristic of the formation of α-helical secondary structures, are observed only above pH 9. At pH 11, the ratio *Θ*_222_/*Θ*_208_ approaches 1, indicating that the helical peptides assemble predominantly into stable coiled-coil homodimers. Measurements were performed in 10 mm sodium phosphate and at a peptide concentration of 65 μm. Inset shows *Θ*_222_/*Θ*_208_ versus ionic concentration, demonstrating that helical peptides form at pH 3 with NaCl concentrations ≥0.5 m, with stable coiled-coil homodimer assembly dominating at 2.5 m NaCl. Measurements were performed in 10 mm sodium phosphate at pH 3.

The well-defined interactions that drive the formation of helical secondary structure, coupled with the ability to introduce chemoselective moieties for oriented immobilization, make BASE-C an excellent exemplar peptide to investigate the effect of surface immobilization and molecular crowding on molecular conformation. Peptide monolayers were formed on copper-ion-functionalized quartz slides onto which BASE-C can chemisorb through the C-terminal cysteine (see [13] and SI for details). All CD experiments were performed using two quartz slides with peptide monolayers formed on both sides and mounted within the measurement cuvette. The use of multiple surfaces not only increases the signal-to-noise ratio but also decreases alignment of the immobilized polypeptides. Baseline CD spectra of the copper-ion-functionalized quartz surfaces (Figure S3) were subtracted from measurement data.

In contrast to solution-phase measurements, which showed a random-coil conformation at pH 7, on-surface CD spectra of the immobilized BASE-C peptides show pronounced CD minima at 222 nm and 208 nm characteristic of α-helical secondary structure (Figure 2). We note that the CD band at 208 nm is influenced by orientation of helical peptides relative to the direction of light propagation.[14] Although the four functionalized surfaces were not intentionally aligned, the surface-immobilized peptides are likely to be more oriented than in solution, which could reduce the intensity of the CD band at 208 nm. However, given that we observe a pronounced double minimum, in contrast to a broad single minimum that results from CD dominated by alignment effects, we conclude that the alignment plays a secondary role. An identical α-helical structure was also observed using BASE-C immobilized onto a gold surface (Figure S4). Furthermore, a random-coil peptide immobilized on copper-ion-functionalized quartz slides was found to remain in a random-coil conformation (Figure S5). These experiments suggest that the folding of BASE-C into a helical conformation is due to intermolecular interactions between immobilized peptides rather than an artifact of the experimental approach.

**Figure 2:**
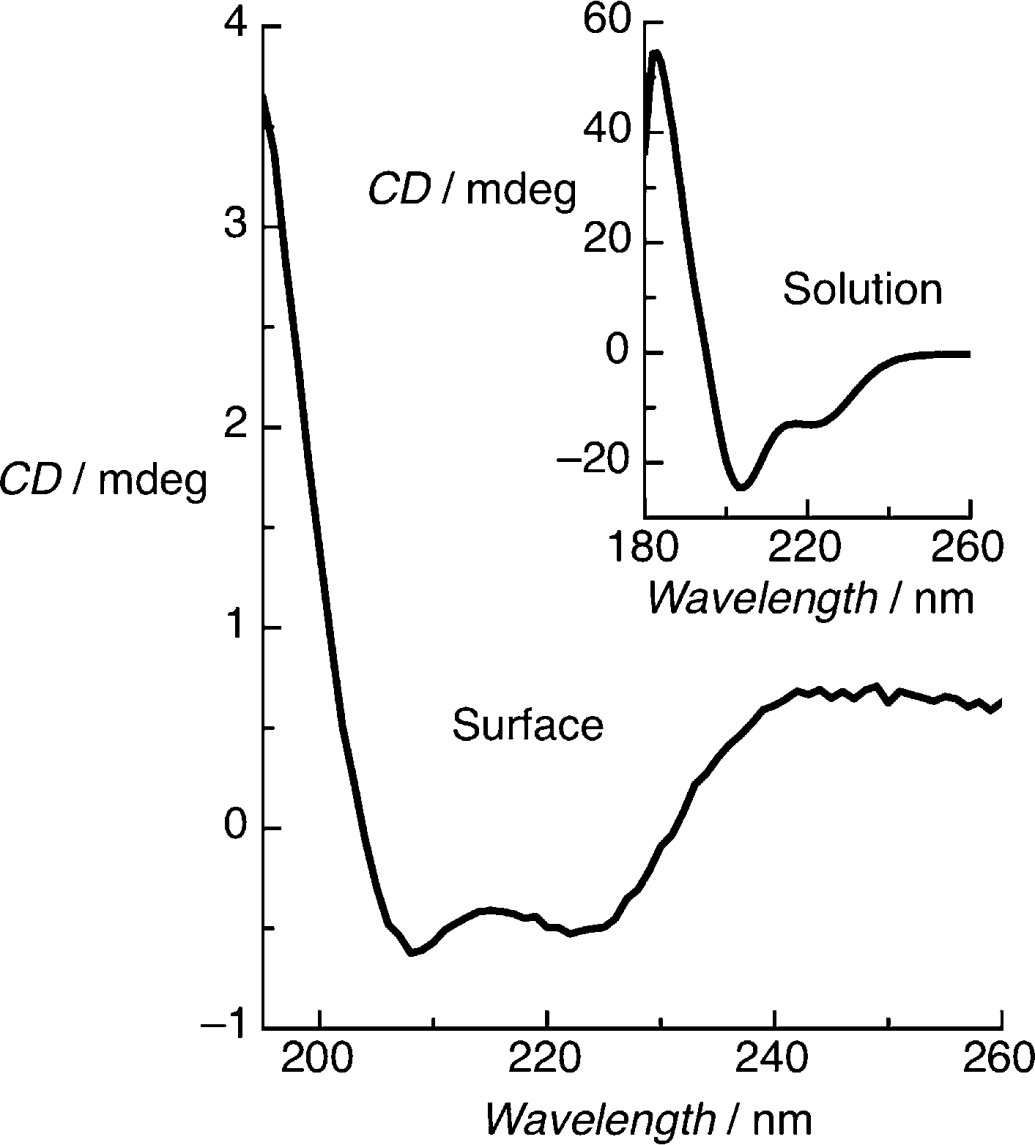
On-surface CD spectrum of a BASE-C monolayer immobilized on a copper-ion-functionalized quartz surface at pH 7. Inset: Characteristic solution-phase CD spectrum of BASE-C in solution at pH 7 showing random-coil conformation. CD measurements were performed in 10 mm sodium phosphate.

From the signal intensities of the on-surface CD spectra we estimate the surface density of immobilized peptides to be 3.3×10^13^ peptides per cm^2^ (see SI). This is comparable to the surface density of a monolayer of alkanethiolates assembled using the same surface chemistry[13] and suggests BASE-C forms a highly packed peptide monolayer. Finally, assuming a perfectly packed layer of cylindrical peptides assembled perpendicular to the surface, we calculate a peptide diameter of less than 2 nm, which compares well with the diameter of a coiled-coil dimer.[15]

Solution-phase measurements showed a strong dependence between pH and molecular conformation, with the α-helical conformation being favored under increasingly basic conditions. We also observe a qualitatively similar behavior following the immobilization of BASE-C, as shown in Figure 3. Critically, the threshold pH at which the peptides switch between random-coil and α-helical conformations differs markedly between solution-phase and surface-immobilized peptides. Specifically, immobilized BASE-C is observed to adopt an α-helical conformation in solutions with a pH>4 compared to pH 9 for the solution-phase system.

**Figure 3:**
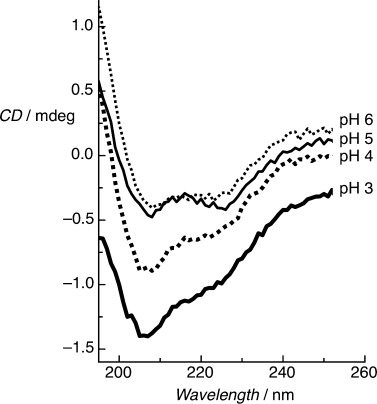
On-surface CD spectra of immobilized BASE-C peptide at different pH. CD minima at 222 nm and 208 nm, typical of the formation of an α-helical secondary structure, are observed above pH 4 only. Below pH 4 the immobilized peptides adopt a random-coil conformation.

The electrostatic charge can be screened in solution by increasing the ionic strength such that the hydrophobic effects, which drive the assembly of the stable coiled-coil homodimer, dominate even at pH 3 (Figure 1). This dependence on ionic strength is also observed for surface-immobilized BASE-C (Figure 4) although the ionic strength at which screening occurs is significantly lower than in the solution-phase (50 mm versus 1.5 m, respectively). We note that the difference in ionic strength between pH 3 and pH 7 is approximately 10 mm.

**Figure 4:**
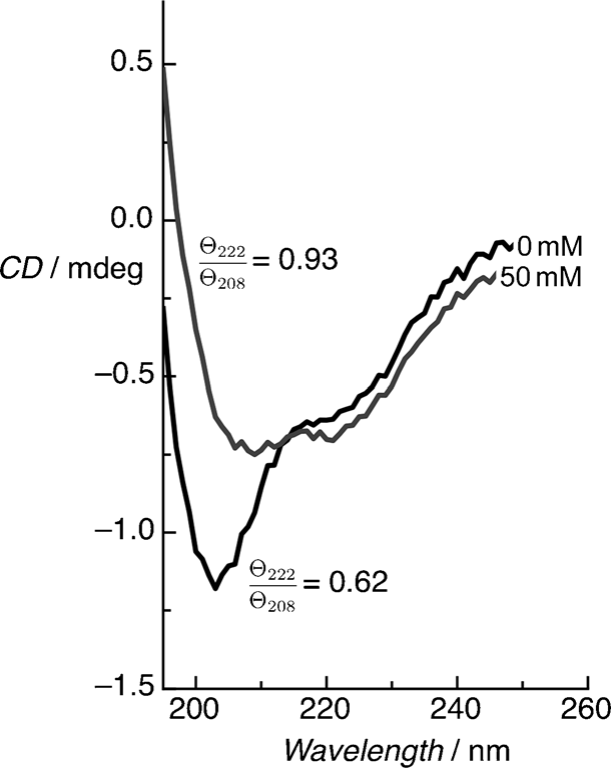
On-surface CD spectra for the immobilized BASE-C peptide at pH 3 for different NaCl concentrations. Screening of repulsive electrostatic interactions at higher ionic concentrations (>50 mm) promotes increased folding of the peptide into a stable, helical conformation even at pH 3 as indicated by the increase in *Θ*_222_/*Θ*_208_ ratio.

As with BASE-C in solution, these pH and ionic strength dependencies suggest that peptide folding is regulated by the interplay between electrostatic and hydrophobic effects. Assuming that the peptides are α-helical and oriented, we calculate a peptide concentration in the monolayer of around 100 mm; significantly higher than the solution-phase peptide concentration (65 μm). This, together with the fact that conformational change occurs at significantly reduced pH and ionic strength, indicates that molecular crowding plays a significant role in governing the conformation of surface-immobilized peptides. The dependence between peptide concentration and BASE-C conformation can also be observed in solution (Figure 5). Critically, the pH at which the peptide changes between random coil and α-helical conformations is found to be reduced with increasing peptide concentration. Immobilization of BASE-C through thiol chemistry effectively forces the system into this high (surface) concentration regime in which hydrophobic effects drive folding into an organized, α-helical monolayer.

**Figure 5:**
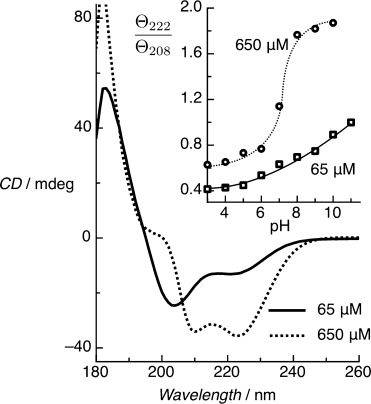
Solution-phase CD spectra for the BASE-C peptide at pH 7 for two different peptide concentrations. The assembly of stable coiled-coil homodimers, indicated by *Θ*_222_/*Θ*_208_≥1 (see inset), occurs at pH 7 when the concentration of peptides in solution is 650 μm. In contrast, for a 65 μm solution we only observe *Θ*_222_/*Θ*_208_≥1 above pH 11.

To understand the spatial organization of peptides within the immobilized BASE-C film in more detail, we performed molecular dynamics (MD) simulations of a BASE-C monolayer assembled on gold from 24 individual peptides with an assumed α-helical conformation (Figure 6 a).[16–18] The average monolayer thickness, measured from the sulfur moiety to the C atom of the methyl group in the final alanine, was 42.6 Å, whereas the average distance between adjacent sulfur moieties was found to be 15.1 Å. This is equivalent to a surface density of 4.4×10^13^ peptides per cm^2^ and compares well with the surface density derived experimentally. The peptides are organized into a well-defined, crystalline monolayer, as shown in Figure 6 b, driven by hydrophobic interactions that force the hydrophobic leucine residues exposed on the helical peptide surface to aggregate, screening them from the surrounding solvent. This leads to the formation of a repeating pattern of well-defined hydrophobic and hydrophilic regions.

**Figure 6:**
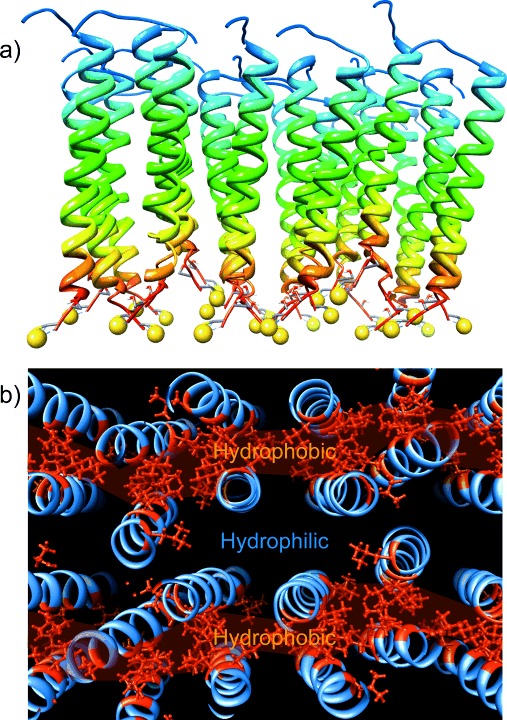
Representative snapshots of MD simulations of a) side view and b) top view of a monolayer assembled on Au from 24 individual BASE-C peptides. The yellow spheres are sulfur moieties associated with the cysteine residue. The gold surface has been removed for clarity. Part (b) also plots the organization of hydrophobic leucine residues (highlighted in red) within the peptide monolayer.

In conclusion, surface-immobilized systems can provide unique insights into the effect of molecular crowding on the conformation of peptides and proteins in high-molecular-density environments, and biophysical tools are today available for measuring molecular conformation on-surface. Here, we used on-surface CD to study an immobilized exemplar peptide and show that molecular crowding, which results directly from immobilization, dramatically shifts the pH and ionic strength at which the peptides undergo a structural transition between random coil and α-helical conformation. Although the kinetics and thermodynamics governing the interaction between an immobilized protein and a ligand in solution are well established, our results show that interactions between proteins in an immobilized layer can also strongly influence molecular conformation and need to be considered, for example, using on-surface CD or complementary tools such as IR spectroscopy, in the design and interpretation of experimental studies involving immobilized molecular systems.
