# Supplementary material for: The Influence of Two-Dimensional Organization on Peptide Conformation
Source: Angew Chem Int Ed Engl. 2014 Nov 20;54(3):974–8. doi: 10.1002/anie.201408971 (PMC4506555; doi:10.1002/anie.201408971)
Supplement: Supplementary file 1 [file anie0054-0974-sd1.pdf]

Angewandte Chemie
Eine Zeitschrift der Gesellschaft Deutscher Chemiker

Supporting Information

© Wiley-VCH 2014

69451 Weinheim, Germany

The Influence of Two-Dimensional Organization on Peptide Conformation**

Simon J. White, Steven D. Johnson, Mark A. Sellick, Agnieszka Bronowska, Peter G. Stockley, and Christoph Wälti**

anie_201408971_sm_miscellaneous_information.pdf

Contents:

Experimental section
Supporting Figures S1 – S5

Experimental Section

Materials

BASE-C (AQLKKKLQANKKKLAQLKWKLQALKKKLAQGGGSC) and BLK (SLDTLAEQLDPSANNVLSC) peptides were purchased from PeptideSynthetics/Peptide Protein Research Ltd (Bishops Waltham, U.K.). All other chemicals were purchased from Sigma-Aldrich (Dorset, U.K.)

Solution-phase Circular Dichroism

Measurements were performed on a Chirascan (Applied Photophysics) in 1 mm quartz cells. Scans were made from 180 to 260 nm at a scan rate of 1 nm/second. Peptides (0.25 mg/mL which corresponds to 65 μM) were dissolved in 10 mM sodium phosphate buffer (outside of pH 4 and 9, HCl or NaOH were used to attain the correct pH). An average of three scans was used and a buffer blank was subtracted from the raw data. The CD spectra of BASE-C with increasing NaCl concentration can be seen in figure S1.

On-Surface Circular Dichroism

Measurements were performed as in the solution-phase experiments except a custom cuvette (Figure S2) from Hellma analytics (Müllheim, Germany) was used. Two prepared slides (details of surface functionalization given below) were placed in the cuvette in the appropriate buffer (note that the cuvette has room for five slides). Due to the low numbers of peptides an average of 10 scans was needed to reduce noise in the data to acceptable levels. For changes in buffer, i.e. salt or pH, the slides were washed three times in the new buffer and then incubated overnight in the desired buffer.

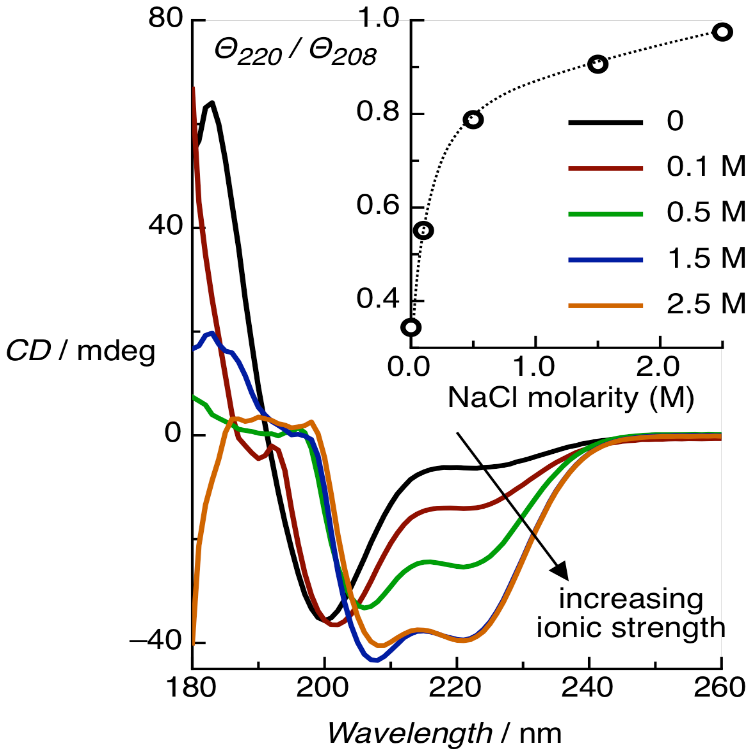

Figure S1. CD spectra of BASE-C (0.25 mg/mL) in 10 mM sodium phosphate pH 7 with increasing NaCl concentration.

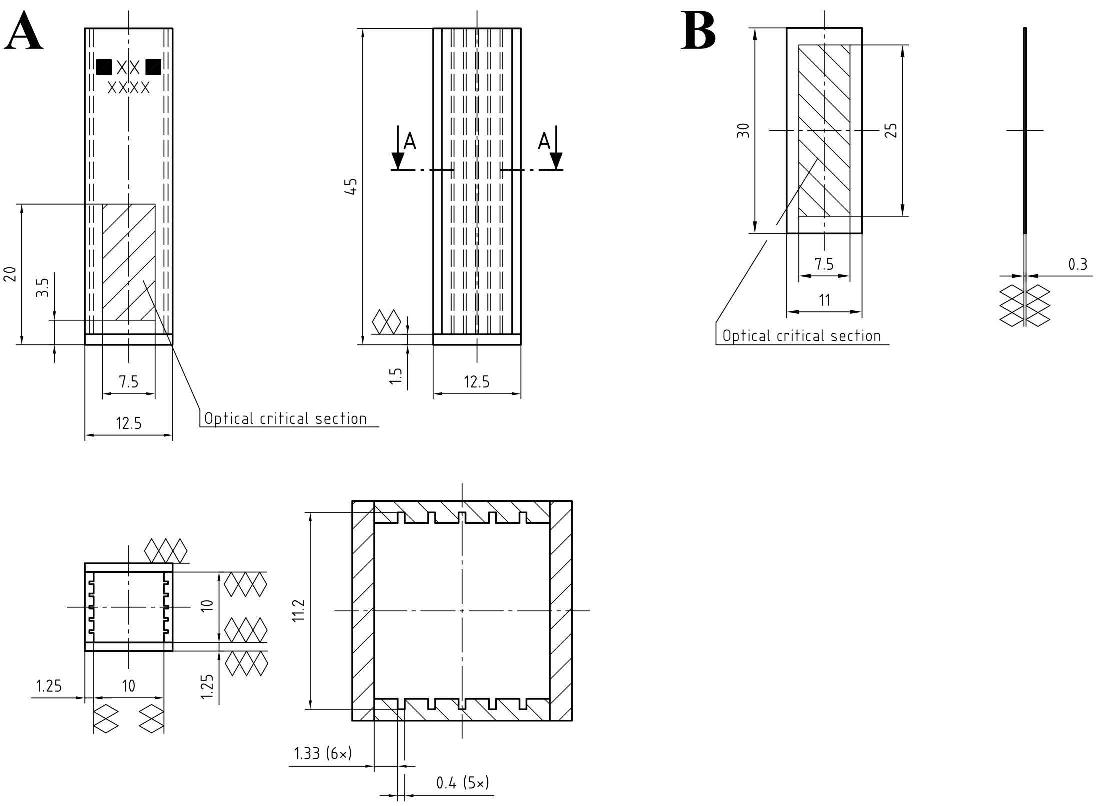

Figure S2. Schematics of (A) the custom circular dichroism cuvette used in this study

and (B) the slides. Hashed areas show the areas that were measured by the CD spectrometer. Images are shown with the permission of Hellma analytics.

To prepare a buffer blank the slides were scanned in the presence of buffer before the peptide was added (see below). Therefore surfaces had the silane linker and copper present to act as a blank (an example of the scan can be seen in Figure S3).

Peptide data was corrected by subtracting the average buffer blank spectra from the average peptide scan spectra.

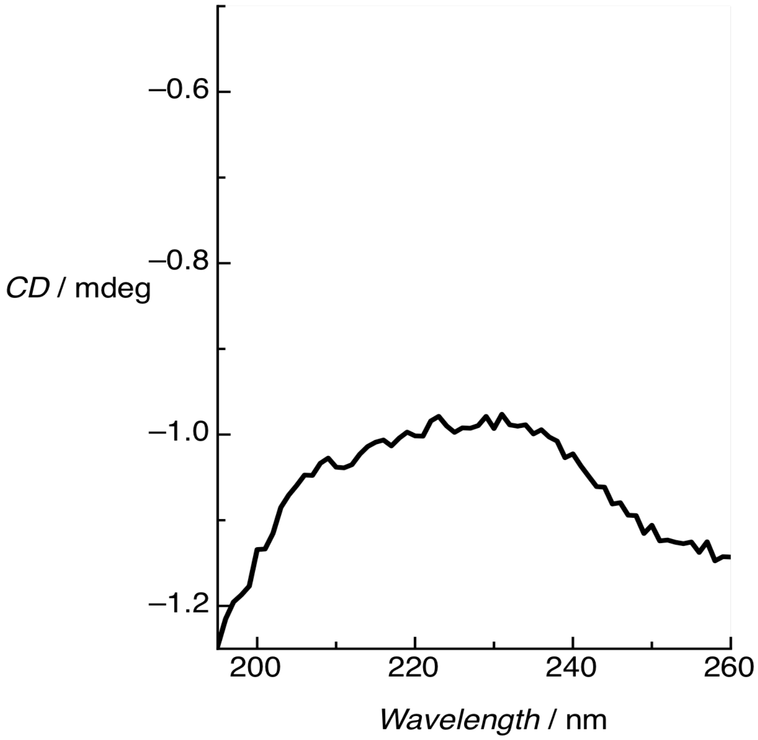

Figure S3. CD spectra of quartz surface before peptide immobilization in the presence of 10 mM sodium phosphate buffer pH 7.

Preparation of gold-coated quartz surfaces for the custom Hellma analytics cuvette

The slides were cleaned as below but after the silane layer had been formed overnight, the slides were washed in 100% isopropanol and 100% ethanol (slides were placed in 15 mL falcon tubes containing these solutions for 5 minutes). After careful drying, a 10 nm layer of gold was evaporated directly onto the mercaptosilane functionalized removable side of a quartz cuvette using an Edwards 306A thermal evaporator. Once again, peptides were immobilized on the surface by incubating the slides in a 15 mL falcon tube containing 6 mL of 0.1 mg/mL peptide in sodium phosphate pH 7. After incubating overnight the slides were washed 5 times in 10 mL of sodium phosphate buffer pH 7. A CD scan of the peptide-coated gold surface can be seen in Figure S4.

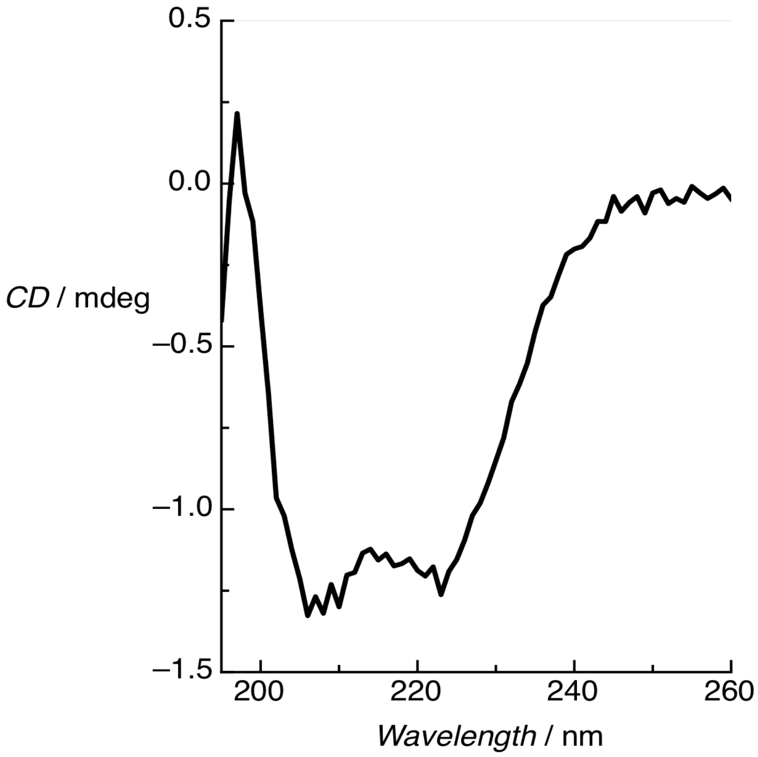

Figure S4. CD spectra of BASE-C immobilized on to a gold surface.

Preparation of quartz surfaces for the custom Hellma analytics cuvette

Each quartz slide from Hellma was washed in 100% acetone for 10 minutes with sonication, followed by 10 minutes in 100% isopropanol, also with sonication. After 5 minutes in each wash the slides were turned over to ensure both sides were cleaned of impurities. The slides were then dipped in ultrapure water before being placed into Piranha solution (30% H_2O_2, 70% H_2SO_4) for 10 minutes after which time the slides were turned over and incubated for a further 10 minutes. The slides were then sonicated for 10 minutes in ultrapure water, 30 minutes in 100% ethanol and 30 minutes in 100% isopropanol (once again turning the slides over after half the time had elapsed).

After cleaning, the slides were placed in a 15 mL falcon tube containing 8 mL of a 4% (3-mercaptopropyl)trimethoxysilane solution in isopropanol and incubated overnight at room temperature. Using the 15 mL falcon tube ensured that both sides of the slide were in contact with the silane solution.

The slides were then washed in 100% isopropanol and 100% ethanol (slides were placed in 15 mL falcon tubes containing these solutions for 5 minutes). They were then incubated in 1 mM copper(II) perchlorate hexahydrate (98%) in ethanol for 10 minutes. After incubation the slides were washed (as above) in 100% ethanol and finally sodium phosphate buffer (pH 7). Peptides, either BASE-C or BLK (an example of a CD spectra of the BLK peptide can be seen in figure S5) were immobilized on the surface by incubating the slides in a 15 mL falcon tube containing

6 mL of 0.1 mg/mL peptide in sodium phosphate pH 7. After incubating overnight the slides were washed 5 times in 10 mL of sodium phosphate buffer pH 7.

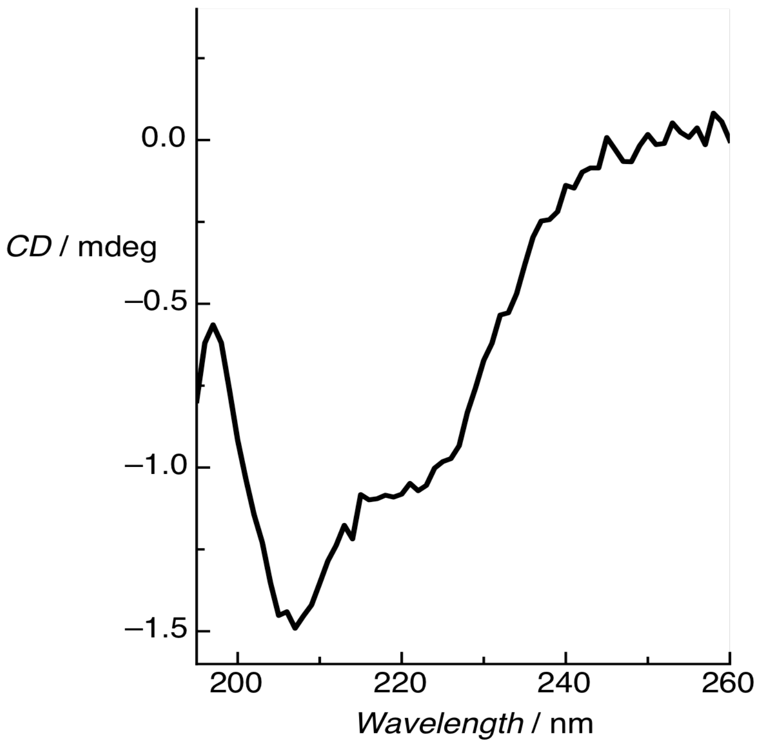

Figure S5. CD spectra of immobilized BLK peptide on a quartz surface.

Estimation of solution concentration and surface density of BASE-C peptide

The interaction volume used in solution-phase CD measurements was estimated to be 1 μL (1 mm^2 light beam and a 1 mm-pathlength CD cuvette). At a solution concentration of 65 μM the interaction volume contained 3.9×10^{13} peptides. The on-surface CD signal intensity is approximately 30 times lower than the equivalent solution-phase measurement (at 208 nm). Using this 30-fold decrease in signal intensity and assuming identical surface density on the two functionalized quartz slides (i.e. four functionalized surfaces) we estimate a surface packing density of 3.3×10^{13} peptides/cm^2.

MD simulation protocol

The BASE-C peptides were attached to the Au surface as described in Johnson *et al.* [13]. In our simulations, the peptides were covalently connected to the Au atoms of the surface via the C-terminal cysteine residue [13]. The control simulation contained 24 peptides in aqueous solution (no surface). In order to study the role of the crowding on peptide dynamics and folding, another simulation setup consisting of only 3 BASE-C peptides immobilised on the Au surface was studied.

The BASE-C peptide were initially in unfolded conformations (random coil) for all simulations. For the simulation of the surface-immobilized peptides, the simulation box contained 24 peptides, and the dimensions of the Au surface was approximately 8.8 nm x 9 nm.

All systems were investigated at neutral pH (lysine side chains were protonated), and were immersed in TI3P water. Chloride ions were added to the systems to maintain neutrality. Prior to MD simulations, the systems were energy-minimised by 2500 steps of steepest-descent, followed by 5000 steps of conjugated-gradients molecular mechanical calculations.

MD simulations were carried out with the AMBER11 package [16], with Amber99SB parameters for peptides and ions [17]. The parameters used for Au surface were as described in Johnson *et al.* [13]. The simulations were carried at 300 K, at constant pressure (1 bar), for 70 ns, with time steps of 2 fs. The cutoff for non-bonded interactions was 12 Å. The coordinates were saved every 1 ps. The analysis was performed by ptraj module [16], and the results were visually inspected using the UCSF Chimera package [18].
